# Extracellular Vesicles as New Players in Cellular Senescence

**DOI:** 10.3390/ijms17091408

**Published:** 2016-08-26

**Authors:** Lorena Urbanelli, Sandra Buratta, Krizia Sagini, Brunella Tancini, Carla Emiliani

**Affiliations:** 1 Department of Chemistry, Biology and Biotechnology, University of Perugia, Via del Giochetto, 06123 Perugia, Italy; sandra.buratta@unipg.it (S.B.); krizia.sagini@studenti.unipg.it (K.S.); brunella.tancini@unipg.it (B.T.); 2 Centro di Eccellenza sui Materiali Innovativi Nanostrutturati (CEMIN), University of Perugia, Via Elce di Sotto 8, 06123 Perugia, Italy

**Keywords:** senescence, aging, senescence-associated secretory phenotype (SASP), extracellular vesicles, exosomes, ectosomes, microvesicles

## Abstract

Cell senescence is associated with the secretion of many factors, the so-called “senescence-associated secretory phenotype”, which may alter tissue microenvironment, stimulating the organism to clean up senescent cells and replace them with newly divided ones. Therefore, although no longer dividing, these cells are still metabolically active and influence the surrounding tissue. Much attention has been recently focused not only on soluble factors released by senescent cells, but also on extracellular vesicles as conveyors of senescence signals outside the cell. Here, we give an overview of the role of extracellular vesicles in biological processes and signaling pathways related to senescence and aging.

## 1. Introduction

Cellular senescence is a complex biological phenomenon that has raised considerable interest in recent years. Cells respond to environmental insults not only by dying (“necrosis”) or programming their death (by “apoptosis”, “autophagy” or “programmed necrosis”) [1], but also by entering into a state of permanent proliferation arrest called “cellular senescence” [2]. At first sight, senescence can be seen as a safety mechanism allowing organism to arrest the proliferation of damaged cells, which would otherwise proceed towards oncogenic transformation. Therefore, it represents a barrier towards neoplastic lesions [3]. However, senescence can also become a double-edge sword, as cells that have undergone permanent proliferative arrest can be potentially detrimental for the whole individual: if these cells are not promptly cleared by immune system, similarly to apoptotic cells, their accumulation can either lead to aging or promote cancer, depending on the tissue context [4]. The presence and age-dependent accumulation of senescent cells in aged tissues has been demonstrated [5], and convincing evidence has been provided that such accumulation can accelerate the decline of tissue functions and promote age-associated diseases [6,7,8]. In addition to this well appreciated role in aging and tumor suppression, recent findings have shown that senescence also plays a pivotal role in the tissue remodeling associated with embryonic development and wound healing [9]. 

The lifespan prolongation in the Western world, together with the consequent increase in the prevalence of neurodegenerative and metabolic disorders associated with aging, is challenging public health systems of industrialized countries. For this reason, investigation of cellular senescence as a key mechanism, whose elucidation can lead to the development of specific anti-cancer as well as pro-healthy aging therapies, has expanded over recent years. 

Here, we review current knowledge on the involvement of extracellular vesicles in biological processes and signaling pathways related to cellular senescence and organism aging.

## 2. Cellular Senescence

Senescence has been initially characterized in vitro as a process limiting the proliferative capability of primary cells [10]. The proliferation arrest, which is the main feature of cell senescence, is accompanied by morphological as well as functional changes. From a morphological point of view, senescent cells are characterized by an enlarged, flattened morphology, extensive vacuolation and increased autofluorescence, due to the accumulation of undegraded macromolecules [11,12]. In addition, these cells show positive staining to senescence-associated β-galactosidase (SA-βGal), which is defined as β-galactosidase activity detectable at pH 6.0 and is one of the most used indicators of senescence [13]. Interestingly, despite its identification as lysosomal β-galactosidase [14] and its diffuse experimental use, the molecular mechanism underlying its application as senescence biomarker has not yet been clarified [15].

During the last few decades, it has become clear that different stimuli can induce cell senescence. In particular, DNA damage appears to be a key point for the induction of this phenotype [16]. As a matter of fact, many physical and chemical stressors, arising both intracellularly and extracellularly and known to cause DNA damage and genomic instability, actually induce cell senescence, such as UV irradiation, reactive oxygen species and mutagenic substances [17]. Telomere shortening, which ultimately affects genome integrity, is another well characterized cause of cellular senescence, possibly the most prevalent cause of the proliferation arrest of primary cell cultures discovered by Hayflick [6]. 

Besides direct DNA damage, the activation of oncogenes has been also discovered to prompt cellular senescence, leading to the definition of peculiar senescence process termed oncogene-induced senescence (OIS) [18]. How can oncogenic signals, which are implicated in the development of a pathology characterized by deregulated proliferation, actually induce senescence, whose main feature is proliferation arrest? Paradoxically, oncogene activation induces senescence at least in part by inducing DNA damage, leading to the persistent activation of DNA damage response (DDR) signaling detected in senescent cells [19], although in some cases DDR-independent mechanism have been proposed [3]. 

Whatever the initiating stimulus, cell senescence is mainly controlled by the p53/p21 and p16INK4a/pRB pathways. Therefore, maintaining the integrity of these two networks is crucial for the establishment of the senescent phenotype, as shown by multiple studies in which the inactivation of p53 or pRB not only prevents senescence associated growth arrest, but also increases neoplastic transformation [20]. Stimuli leading to DDR (as for example, ionizing radiation and telomere dysfunction) prompt the transcriptional activation of p53, which in turn induces the expression of genes inhibiting cell cycle progression. In particular, the cyclin-dependent kinase inhibitor (CDKI) p21 is the main mediator of p53-dependent senescence. Among other activities, it suppresses the phosphorylation of pRB, thus providing a connection between the p53/p21 and p16INK4a/pRB networks. The accumulation of p16INK4a has also been found in senescent cells [21], but the molecular mechanisms responsible for p16 upregulation are still poorly understood. p16INK4a is another CDKI that keep pRB in an active, hypo-phosphorylated form, preventing the activity of E2F, a transcriptional factor required for the expression of key genes encoding proteins that stimulate or facilitate cell-cycle progression. Even if both p21 and p16INK4a eventually act on pRB pathway, they are not equivalent, and differences depending on senescence stimulus and cell type have been reported [22]. Moreover, only the p16INK4a/pRB pathway has been shown to be crucial for generating senescent-associated heterochromatin foci (SAHF), local changes in chromatin organization that result in the silencing of genes needed for cell proliferation [23].

## 3. Senescence Associated Secretory Phenotype

Cellular senescence is not only an in vitro process but it has also in vivo implications, as it is largely appreciated that the number of senescent cells in tissues correlates with aging and age-related disorders [24]. However, it is not clear if the age-dependent increase of senescent cells is due to a higher presence of cells with damaged DNA or to a deterioration of the tissue capability to remove such cells using immune system macrophages and to replace them with stem cell progenitors that undergo differentiation [11].

How do senescent cells contribute to aging in the context of a tissue or organism? The best candidate to play a pivotal role in transmitting signals from senescent cells to the surrounding tissue is the so-called senescence-associated secretory phenotype (SASP). Indeed, senescent cells release a peculiar secretome, whose components include cytokines (interleukin (IL)-1β, IL-6), chemokines (IL-8, and monocyte chemoattractant protein-1 (MCP-1)), growth factors (transforming growth factor β (TGFβ), basic fibroblast growth factor (FGF), hepatocyte growth factor (HGF), and vascular endothelial growth factor *(*VEGF)) and extracellular proteases such as matrix metalloproteinases [25]. Besides, remodeling of extracellular matrix by secretion of peculiar collagen and proteoglycan species has also been found [9,25]. 

SASP components can facilitate the removal of senescent and/or the remodeling of tissue by attraction of phagocytic immune cells, which clean up these cells. However, this removal process can in turn generates a strong pro-inflammatory microenvironment capable of promoting tumor initiation and progression. Indeed, several of the secreted cytokines and chemokines have a well-established pro-inflammatory role (IL-6, and IL-8), thus suggesting that SASP may be one of the players of the so-called inflammaging, i.e., the age-associated pro-inflammatory phenotype that is involved in the development of age-associated diseases [26]. On the other hand, SASP components may stimulate the proliferation of surrounding cells as part of a global response, which communicate to neighbor cells their compromised state in order to stimulate tissue repair [9], as for instance the need of new vessels through the release of VEGF [27]. Again, this stimulation of cell growth can have detrimental consequences, promoting the proliferation of pre-neoplastic cells harbored in the tissue, or inducing the epithelial-to-mesenchymal transition in susceptible cells [28].

Interestingly, there is a certain consensus that key members of the Wnt pathway are SASP components [29]. Furthermore, an enhanced Wnt signaling in a mammalian model of accelerated aging has been reported [30]. The Wnt pathway plays a critical role during embryo development and tissue regeneration. However, how Wnt proteins travel in the extracellular space has remained a largely unresolved question for years. The reason is that the palmitoylated Wnt proteins are membrane-bound and unlikely to be released as soluble proteins into the extracellular space. However, recently, Wnt proteins have been shown to be directly secreted, not as soluble factors, but as a part of small vesicles released extracellularly termed exosomes. These vesicles carrying Wnt proteins on their surface have been reported to activate Wnt signaling in target cells [31,32]. The finding has shed a new light on the role of extracellular vesicles (EVs) in mediating the cell-to-cell transmission of senescence signals, suggesting that EVs represent a new, multi-faceted, and still poorly characterized component of SASP.

## 4. Extracellular Vesicles (EVs)

The discovery that cells release in the extracellular environment different types of vesicles is changing our knowledge of cell-to-cell-communication. In fact, in addition to signaling mediated by soluble factors, it has emerged over the last two decades that cells also communicate by releasing vesicles that can act on nearby cells (paracrine signaling) or end up in circulating body fluids influencing distant sites (endocrine signaling), although this last aspect has been much less characterized [33].

EV is a general term to indicate membrane surrounded structures released outside the cell. Currently, these vesicles have been mainly characterized on the basis of their cellular origin and dimension. Based on their origin, EVs can be divided into exosomes, originating from the endosomal compartment, and microvesicles, budding from the cell membrane [34], which are also referred to as ectosomes [35]. A third type of extracellular vesicles is often included in this list, i.e., the apoptotic bodies, which are released upon fragmentation of apoptotic cells and are characterized by a larger size (500–5000 nm) [36]. In more detail, exosomes originate from the membrane invagination of a subset of late endosomes, which end up containing a large number of small vesicles and take the name of multi vesicular bodies (MVB). Exosomes are characterized by small dimensions (30–100 nm) and round shape. Instead, microvesicles or ectosomes bud from the plasma membrane and are characterized by a wider size distribution (from 100 nm to 1000 nm), and a less regular morphology. Despite their different origin, which in theory should make easy to distinguish them, the evidence that microvesicles of small dimensions share a similar size and density with exosomes has made their separation on the basis of physico-chemical properties very difficult [37]. 

For this reason, the currently available purification methods, mostly based on ultracentrifugation, can ultimately separate only EVs of small dimensions from those of large dimensions, but cannot guarantee EVs preparation containing only vesicles of endosomal origin or budding from the cell membrane. A further complication for the purification of specific vesicles is the evidence of a dynamic trafficking between the endosomal compartment and the plasma membrane, which makes the presence of the so-called exosomal markers often enriched, but not exclusive, of exosomes [38,39,40,41]. 

The first reason that made EVs so interesting for the scientific community is their biochemical content. EVs contain not only lipids and proteins, but also miRNA and mRNA. More recently, the presence of DNA, such as genomic and mitochondrial DNA, has also been reported [32]. Perhaps the most relevant implication of mRNA and miRNA presence within EVs is their ability to transmit signals to recipient cells. As a matter of fact, in a pioneering paper published in 2007 by Valadi et al. [42], exosomes were shown to deliver specific mRNAs to target cells, where they were also translated into functional proteins. In a similar manner, the miRNAs content of EVs was shown to affect gene expression in recipient cells [43]. Even if molecular details dealing with the packaging of mRNA and miRNA into EVs have not been fully elucidated [44], the EV-mediated exchange of genetic material among cells has provided evidence of a previously unknown mechanism of horizontal transfer of nucleic acids. 

The EVs content of proteins and lipids is also peculiar. These vesicles contain a set of proteins that are common among EVs of different cellular origin, as well as proteins that are typical of the releasing cell. According to the currently available databases centered on EVs content, i.e., Vesiclepedia (www.microvesicles.org) [45], EVpedia (www.evpedia.info) [46] and ExoCarta (www.exocarta.org) [47], the most commonly found proteins in EVs are tetraspannins (CD9, CD63, and CD81), specific stress proteins (heat shock proteins; HSPs), members of the ESCRT complex involved in the biogenesis of EVs (Tsg101, and Alix), and proteins implicated in vesicles trafficking (i.e., Rabs). Although these proteins, namely tetraspannins, have been considered exosome-specific for years, there is now a general consensus that they are only enriched in exosomes, and possibly present only in certain subgroups of vesicles [37].

As for proteins, the lipid composition of EVs is distinct from the cell of origin, but it mirrors the releasing cell type. In addition, several studies have confirmed that a common feature of EVs lipid content is the enrichment in cholesterol and sphingolipids, a composition closely resembling that of lipid rafts, i.e., detergent-resistant microdomains [48]. Furthermore, EVs also contain many lipids that are signaling mediators, and phospholipases involved in the release of these mediators from membrane phospholipids. Like nucleic acids and proteins, EV lipids are able to activate signaling in recipient cells, as it has been shown that vesicle-bound prostaglandins trigger prostaglandin-dependent intracellular pathways [49], and EV endocannabinoids released by microglial cells are able to stimulate type-1 cannabinoid receptors [50]. 

Besides their peculiar content, the second reason that has made EVs so attractive is their ubiquitous presence in body fluids, including those easily accessible for diagnostic purposes. The presence of EVs has been described in blood (plasma, serum), urine, saliva, cerebro-spinal fluid, ascites, breast milk, amniotic fluid, and seminal liquid [34]. Furthermore, EVs maintain the biochemical features of releasing cells, eventually including markers typical of their pathological state. As a consequence, they appear a potentially minimally invasive source of biomarkers for the diagnosis and prognosis of several pathologies, and many investigations have addressed this issue [51,52].

## 5. The Release of EVs in Cell Senescence and Aging

Many studies have shown that the secretion of EVs is enhanced in cells undergoing stress conditions. In particular, alteration of intracellular calcium level and changes in the synaptic activity have been shown to prompt EVs release [53,54]. Furthermore, cisplatin and other chemotherapeutic treatments resulted in an increased release of vesicles in vitro [55]. Although it is not clear why cells undergoing stress would release more vesicles or vesicles with different content, a possible explanation is that cells release EVs to dispose toxic and/or unnecessary substances. However, it is also feasible that the main purpose of EVs secretion is to communicate their state to surrounding cells. More likely, the release of EVs can be actually a cellular mean to simultaneously dispose what is not needed and communicate their stress to the surrounding tissue.

In a pioneering paper published in 2008, Lehmann et al. reported [56] that clinically relevant doses of irradiation induced premature senescence rather than apoptosis in human prostate cancer cells. This phenotype was associated with a significant increase of exosome-like vesicles secretion in the extracellular environment. In addition, the release of these vesicles was dependent on p53 activation. The role of p53 in EVs secretion was in agreement with other investigations reporting the regulation of exosome release as a novel function of p53 protein [57]. The transcription of tumor suppression-activated pathway 6 (TSAP6) gene, which is implicated in exosome biogenesis, is also controlled by p53 activation [58]. Besides, p53 was shown to be a transcriptional regulator of other endosomal compartment genes relevant for vesicles biosynthesis [59,60]. Taken together, these observations indicate that senescence consequent to DNA damage induces a p53-dependent increase of EVs biogenesis. More recently, Beer et al. [61] too investigated the secretome of irradiated cells, in this case peripheral blood mononuclear cells (PBMCs). They showed that ionizing radiation induced not only the expression of proangiogenic factors but also the secretion of EVs. Evidence was provided that vesicles and proteins were the two major biologically active components released, as they were able to enhance cell mobility, in particular fibroblast and keratinocyte cell migration, thus mediating the regenerative properties of the cell-free secretome.

Signaling pathways activated in a paracrine manner by senescent cell released EVs are far from being elucidated. However, in a recent study Effenberger et al. [62] investigated how transmembrane proteins are secreted from senescent cancer cells, showing that the release from cell surface of the epidermal growth factor receptor (EGFR) ligand amphiregulin and the tumor necrosis factor (TNF) receptor I is mediated by ADAM metallopeptidase domain 17 (ADAM 17) protease via ectodomain shedding, whereas the release of intercellular adhesion molecule 1 (ICAM1), which is a ligand for lymphocyte function-associated antigen-1 (LFA-1) receptor on leukocytes, is due to microvesicles shedding. 

Another event that is correlated with aging is the deposit of toxic proteins, which is tightly associated with the development of age-associated neurodegenerative disorders, such as Alzheimer’s and Parkinson’s diseases [63]. Nowadays, it is well recognized that EVs may represent not only vehicles by which toxic protein aggregates or their precursors are propagated [64], but also elements crucial for the formation of amyloid deposits and important for the maintenance of the equilibrium between soluble oligomers, which are toxic, and insoluble aggregates, which are not toxic [65,66]. In agreement with this finding, Wang et al. [67] reported that the increased mitochondrial DNA damage associated with normal aging alter the functional capacities of the retinal pigment epithelium (RPE) in age-related macular degeneration. The molecular mechanism involves increased autophagy and exosomes release of intracellular proteins by the aged RPE, which contribute to the formation of drusen, an extracellular, amorphous deposit of material in the macula of the retina.

### The Role of EVs Associated miRNA and mRNA in Senescence and Aging 

MicroRNA are short non-coding RNAs (~20 nucleotides long) that regulate gene expression at post-transcriptional level by binding target mRNAs [68]. As one miRNA can regulate up to hundreds mRNA targets, its expression level can affect cellular processes by influencing different regulatory networks. It has been reported that several miRNAs are involved in organismal aging and cellular senescence, either because they target genes belonging to pathways, which are known to play a role in cellular senescence, such as p53/p21 and p16INK4a/pRB [69], or indirectly, because they have been found to be differently expressed during tissue aging [70].

In the case of the p53/p21 pathway, it has been shown that miR-34a and miR-29 can induce cell cycle arrest not because they directly target p53, but because they contribute to its stabilization by targeting proteins relevant for its regulation, i.e., Sirtuin 1 (SIRT1) and protein phosphatase Mg^2+/^Mn^2+^ dependent 1D (PPM1D) respectively [71,72]. Regarding the p16INK4a/pRB pathway, miR-22, miR-29 and miR-30 families have been reported to target this signaling route [73]. In addition, it is widely appreciated that the miR17-92 cluster, along with its paralogs miR-106a and miR-106b, is down regulated in several aging models and can target both p53/p21 and p16INK4a/pRB networks [74]. Interestingly, the expression level of miR-146 has also been shown to be significantly increased in senescent cells compared with proliferating or quiescent ones. Of interest, two important targets of this miRNA are IL-6 and IL-8, which are well characterized SASP components playing a pivotal pro-inflammatory function, thus suggesting a role for miR-146 in the modulation of senescence-associated inflammation [75].

The analysis of miRNAs that are present in EVs released from senescent cells is important because miRNAs could potentially transmit signals to surrounding tissues, both beneficial (i.e., tissue regeneration and wound healing) and/or detrimental (i.e., prompting senescence or stimulating oncogenic transformation of nearby cells). In addition, vesicular miRNAs are interesting in the context of the aging biomarkers search, because they can be easily amplified from a small quantity of a biological sample. As a matter of fact, there is consensus that passage of time alone is not the best measure of aging, so aging biomarkers are needed and extensively searched in order to clearly define aging, and discriminate healthy aging from age-associated pathological processes [76].

As senescence is usually associated with a decline of tissues regenerative potential, which ultimately leads to a decreased capacity to repair cell and organ damage, it is not surprising that a few investigations have focused on the role of vesicular miRNAs in the transmission of signals influencing the age-associated decline of tissue regeneration. Starting from the evidence that circulating vesicles impact on the osteogenic differentiation capacity of mesenchymal stem cells (MSC) in a donor age-dependent manner, Weilner et al. [77] investigated circulating vesicular miRNAs and demonstrated that miR-31 is present at elevated levels in the plasma of elderly and osteoporosis patients. Similarly, in order to shed light on the causes of renal fibrosis in aged tissue, Wang et al. [78] investigated the role of MSC-EVs in the transmission of senescence signals limiting the tissue ability to repair kidney damage. The analysis of miRNAs differential expression in bone marrow MSC-EVs between young or old rats, and the study of their influence on epithelial-mesenchymal transition (EMT), showed that miR-133b-3p and miR-294 were downregulated in EVs from old rats and inhibited TGF-β1-mediated EMT. This suggested that these vesicular miRNAs could actually play a role in aged renal tissue fibrosis.

MicroRNAs released by senescent cells in the extracellular environment via EVs have been reported to prompt senescence in surrounding cells. In particular, Weiner-Gorzel et al. [79] showed that miR-433 mediated chemoresistance to the chemotherapeutic paclitaxel in ovarian cancer cells was due to the induction of senescence, and that high miR-433 expressing cells released miR-433 into the growth media within EVs. This miRNA modulated the tumor microenvironment by inhibiting growth and inducing senescence in nearby cells. On the other hand, miRNA released within EVs have also been reported to suppress senescence in recipient cells. For instance, van Balkom et al. [80] demonstrated that miR-214, which controls endothelial cell function and angiogenesis, plays a dominant role in vesicle-mediated signaling between endothelial cells, as endothelial cell–derived vesicles stimulated migration and angiogenesis in recipient cells, preventing senescence, whereas vesicles from miR-214-depleted endothelial cells failed to stimulate this process. 

The minimally invasive search of aging biomarkers based on miRNA in humans has been the object of a recent work by Machida et al. [81]. Authors reported the microarray analysis of salivary exosome miRNAs from 15 young and healthy individuals, in comparison with 13 old subjects. Evaluation of results identified miR-24-3p as a novel aging biomarker candidate. 

Despite the fact that several studies have been dedicated to miRNA, vesicular mRNAs are also important because they can be translated into pro-senescence proteins in recipient cells. In particular, Mitsuhashi et al. [82] reported that aging enhanced the amount of vesicular cytokine mRNAs released by macrophages stimulated with amyloid peptide Aβ_1–42_. Authors showed that exosomes harvested from human immune cells or directly isolated immunochemically from serum were a rich source of cytokine mRNAs. However, physiological and pathological consequences of age-dependent differences in cytokines secretion level and in vesicle-associated cytokine mRNAs remain to be elucidated.

## 6. The Role of EVs in the Blood

A consistent amount of work carried out on blood cells has provided convincing evidence that cell senescence is actually tightly associated with EVs release. Blood mainly contains vesicles from platelets, erythrocytes, different types of white blood cells, and endothelial cells. As a matter of fact, from an historical perspective, EVs were first described by Johnstone et al. [83] as released by sheep reticulocytes during erythrocytes maturation. In this context, they were suggested to be a new mechanism to get rid of undesired proteins during cell differentiation. Since then, many studies have been carried out in order to characterize EVs released in the blood, in particular with the aim to characterize changes associated with senescence in platelets and erythrocytes, mostly for practical reasons related to the development of the best practices for blood and hemoderivatives storage. In particular, platelets have a life span of only 8 to 10 days in the circulation, and both senescent and activated platelets release EVs, which represent up to 90% of circulating EVs in blood [84]. As it is widely appreciated that senescence and platelets activation during platelet concentrate (PLC) storage promote the release of platelet derived EVs into the surrounding plasma, the characterization of EVs release during PLC storage has been widely used as model system to investigate mechanisms regulating platelets senescence in vivo. 

Pienimaeki-Roemer et al. [85] reported the analysis of the lipid species present in all PLC components (platelets, released PLC-EVs, and plasma) during five days of in vitro storage, which reflects mechanisms that are active during in vivo platelet senescence. First, results showed an increase of PLC-EVs in stored PLCs, which could explain the senescence-dependent loss of platelet cholesterol and glycerophospholipids in PLCs. Accumulation of precursors for eicosanoid biosynthesis (long-chain poly-unsaturated fatty acids and plasmalogens) and of ligands for G-protein-coupled signaling (lysophosphatydylcholine and lysophosphatidic acid) confirmed the potential of newly generated PLC-EVs as carriers of bioactive molecules. In a more recent study, the same authors [86] further investigated the lipidome and extended their investigation to the proteome of EVs released from senescent platelets. According to their findings, features and composition of these EVs resulted highly heterogeneous and were dependent on the platelets stimulus. Authors speculated that such heterogeneous composition was at least in part due to the evidence that platelet MVBs are in the secretory routes of both platelets granules and exosomes. Nevertheless, they were able to show that platelet EVs were carriers of cargo regulating vascular remodeling, with a specific enrichment of apolipoproteins, ceramide and lipid ligands for lysophospholipid receptors. These evidences supported a role for these vesicles in the development of atherosclerosis.

The procoagulant activity of blood EVs, and the changes associated with senescence, have been the object of an interesting paper by Forest et al. [87]. Shed membrane vesicles released upon activation or apoptosis of several blood cell types in response to numerous stimuli are bioeffectors able to modulate several biological functions, including immunity and inflammation, and carry procoagulant activity through phosphatidylserine exposure. In particular, they represent a reservoir of blood-borne tissue factor (TF) activity, the initial activator of the extrinsic blood coagulation pathway [88]. Authors investigated the procoagulant activity in elderly and young patients under both physiological and pathological conditions by measuring the circulating level of membrane vesicles originating from platelets, endothelial cells and erythrocytes. In elderly patients, they observed a reduced basal level of circulating vesicles with preserved procoagulant activity, as compared with young patients. In addition, they observed that infection was associated with a decrease in both circulating vesicles and their procoagulant activity, which was more pronounced in young than in elderly patients. Similar results were reported by Karlaftis et al. [89], that investigated the procoagulant phospholipid activity of circulating membrane vesicles in healthy neonates, children and adults. Results showed significant age-specific differences in the clotting time, as it was increased in neonates and decreased in children aged 1–16 years, thus indicative of a decreased and increased concentration of membrane vesicles in these populations, respectively.

Another aspect of blood investigations regarded vesicles formation as an integral part of the physiological erythrocyte aging process. Bosman et al. [90] isolated erythrocyte-derived vesicles from plasma by fluorescence-activated cell sorting, analyzed their proteome by mass spectrometry, and compared it with the membrane proteomes of erythrocytes that were separated according to cell age. This approach provided evidence that erythrocyte aging process harbors a specific, band 3-centered mechanism for vesicles generation. The aging-associated increase in erythrocytes membrane concentration of proteasome and small G proteins confirmed that protein breakdown and vesicles formation are intimately involved in the removal of damaged components. Taken together, these data indicated a decrease of the interaction between cytoskeleton and cell membrane as a central event in erythrocyte aging, which prompted membrane budding and vesicles formation.

The direct investigation of the regenerative potential of plasma-derived EVs has been the object of a study by Weilner et al. [91], which reported that vesicles isolated from young donors enhance osteoblastogenesis in vitro compared with elderly-derived EVs. The analysis of their differences showed that EVs content of galectin-3 was reduced in elderly-derived vesicles. Further modulation of intravesicular galectin-3 level confirmed a correlation with an altered osteo-inductive potential, indicating that this protein contributes to the biological response of MSCs to EVs.

## 7. Conclusions

Much evidence has focused the attention of the scientific community on EVs as new components of the SASP. Cellular senescence is associated with an augmented release of EVs, and specifically one of the consequences of DNA damage is actually the secretion of EVs mediated by p53 activation. In addition, investigations on blood cells such as platelets and erythrocytes have further confirmed that senescence is associated with an enhanced release of EVs of heterogeneous dimensions. In this case, findings have also suggested that EVs secretion is a manner to eliminate unnecessary components from cells undergoing senescence. The modification of the external microenvironment mediated by EVs, as for soluble SASP factors, can be either favorable, prompting the elimination of senescent cells and the regenerative potential of surrounding cells, or detrimental, promoting local inflammation by recruitment of immune cells and spreading senescence throughout the tissue. However, the characterization of EVs released from senescent cells is still in its embryonic state and, different from soluble cytokines and other factors whose signaling pathways in recipient cells have been relatively well characterized, networks activated in recipient cells by senescence associated EVs are far from being elucidated and need further investigation. 
